# Surface Microanalysis and Sequential Chemical Extraction as Tools for Reliable Environmental Mobility Assessment of Sb and Other Metals

**DOI:** 10.3390/ijerph19159609

**Published:** 2022-08-04

**Authors:** Jéssica Álvarez-Quintana, Almudena Ordóñez, Efrén García-Ordiales, Rodrigo Álvarez

**Affiliations:** Mining Exploration and Exploitation Department, Escuela de Ingeniería de Minas, Energía Y Materiales, University of Oviedo, 13th Independencia St., 33004 Oviedo, Spain

**Keywords:** sequential extraction procedures, antimony, arsenic, surface microanalysis

## Abstract

Sequential extraction procedures (SEPs) are widely used in environmental studies to infer the chemical and/or mineralogical forms of pollutants of concern in soils and sediments. Although there is no general agreement among the scientific community, these methods have shown some limitations, especially those with a lack of objectivity in their interpretation. In this work, a soil sampling campaign was carried out in an area affected by an abandoned Sb mine. Samples (0–15 cm) were carefully prepared and analyzed by an SEP. They were also studied by conventional mineralogical methods (optical and electronic microscopy, both scanning and transmission, with a coupled energy dispersive X-ray (EDX) spectrometer). When comparing the results obtained from both techniques, some discrepancies are highlighted, with As, Cu, Pb, Sb and Zn as elements of concern. For Sb, Cu, Pb and As the predominant fraction (excluding the residual one) is that associated with organic matter (from 8.54 for Sb to 18.90% for Cu). The fractions of pollutants linked to Fe and/or Mn oxides are quantitatively important for As, Pb and Zn (6.46%, 12.05% and 7.43%, respectively) and almost negligible for Cu and Sb. On the contrary, analyses carried out by EDX at a grain scale pointed out that no detectable quantities of the elements of concern were present on the surface of the organic particles. Sb and Pb were always detectable in Fe oxides (up to 1.84 and 5.76%, respectively). Regarding the role of the clayey fraction, the only disagreement between the employed SEP and the microanalysis is in relation to As. Arsenic bound to clay minerals was found to be an order of magnitude lower than As associated with Fe oxides (0.56% and 6.46%, respectively); in contrast, EDX microanalyses showed similar As contents in both groups. Given the objectiveness of EDX microanalysis, these differences should be considered inaccuracies in the interpretation of the sequential extraction results.

## 1. Introduction

Soil pollution due to human activities is an environmental problem of increasing concern. Except for a few remarkable early works [1,2,3,4,5,6,7], most of the research on the environmental effects of this type of pollution has been carried out in the last 25 years. During this time, an enormous and unapproachable amount of research has been carried out in order to assess the effects that the presence of such pollutants exerts on ecosystems.

Concerning metals and metalloids, abandoned mining activities are among the most important primary sources of soil pollution. The toxicity and mobility of these pollutants depend on their chemical forms and their binding state (precipitated, complexed, etc.) since changes in these aspects might induce trace-element mobilization, favoring environmental contamination [8]. There is general agreement about the dual perspective from which this problem should be addressed: the geological/mineralogical/geochemical viewpoint must necessarily be integrated with biochemical and toxicological aspects [9]. The behavior of metals and metalloids that have been the most extensively studied, mainly because of their abundance or high toxicity, is now acceptably well understood. The greatest efforts have conventionally been focused on arsenic, cadmium, chromium, nickel, lead and mercury, although in recent years some other elements, such as selenium and thallium, have received a great deal of attention. Antimony, on the other hand, despite its teratogenic and carcinogenic nature, is not among those whose environmental behavior is better known. There are two main reasons for this: First, it is a relatively uncommon metalloid, and it has not very often generated situations of conspicuous environmental risk. This is the case of old Sb mines, such as Su Suergiu (Italy), Brioude-Massiac (France), Pezinok (Poland), Glendinning (Scotland), Schlaining (Austria) and Xikuangshan (China) [10,11,12,13,14,15,16,17]. Second, its similarity to arsenic leads, in many cases, to assuming a biogeochemical behavior of antimony similar to that expected for the former, not always with adequate justification [18,19,20].

Sb is a chalcophilic metalloid that has four oxidation states (−3, 0, +3 and +5), the two latter being the most usual in low-temperature environments. It is used as a raw material in the electronics industry and for flame-retardant, alloy and battery manufacturing. Sb poses threats to human health due to its toxicological effect, although in uncontaminated media, their background levels are low (values of Clarke–upper crust and soils are 0.2 mg/kg and 0.5–2.6 mg/kg, respectively, according to Gleyzes et al. [8]). The main sources of natural Sb are widespread mineral deposits that are essentially related to hydrothermal events, whereas anthropogenic soil Sb enrichment is usually related to mining and ore processing [21]. Although more than 40 Sb-bearing species are known (see [22,23] for an exhaustive list), the most common primary mineral of Sb is stibnite (Sb_2_S_3_). Stibnite is, in most cases, accompanied by varying amounts of its weathering products or “Sb-ochres” (stibiconite, cervantite, senarmontite and valentinite). It is also important to note that Sb is also an undesirable waste in Cu extractive metallurgy when the primary Cu ores are composed of fahlore (minerals from the tetrahedrite-tennantite solid solution series).

The European Union currently considers Sb a pollutant of priority interest and, at the same time, a strategic/critical raw material of economic importance, at supply risk [24].

## 2. Background Information

### Approach

It is also well known that the estimation of the bioavailability (portion of the contaminant available for absorption by living organisms) plays a critical role in most risk assessment methodologies. Recent research has made widespread use of the so-called sequential extraction procedures (SEPs) to estimate the bioavailable fraction of a given pollutant. These techniques (which have many variants for different elements or compounds) are purely chemical methods inherited from geochemical prospecting. They follow a general protocol of several steps, each one consisting of attacking the sample with extractants of increasing strength, so that the most mobile metals are removed in the first fraction and continue in the order of decreasing mobility, linking the fraction solubilized at each step to a target phase (i.e., exchangeable, bound to Fe/Mn oxides, bound to clay minerals, bound to organic matter and bound to the primary mineral structure [25]). The reliability of the interpretation of SEPs in terms of these modes of occurrence has been criticized since the beginning of their use by many authors [7,25,26,27,28,29,30,31,32,33,34,35,36,37]. The lack of uniformity among the variety of available protocols and reagents and the lack of quality control have also been cited as weaknesses of SEPs. All of the above influence the quantification of the bioavailable fraction and, thus, the results of the risk assessment methods. The authors’ experience with soils polluted by historical mining activities [38] also points to a misinterpretation of the modes of occurrence of various metals according to the different steps in SEPs. In the specialized literature, some recent research addresses this problem from the perspective of As [39,40], Pb [41], Cr-Ni [42] and, Zn-Cd [43].

The goal of this work, by means of a case study, was to carry out a critical comparison between chemical (sequential extraction and acid digestion) and microscopic methods to evaluate the mobility of Sb and other pollutants from mine wastes. In the specialized literature there are similar studies on the behavior of As [38,44], but none related to Sb, which in this case is the pollutant of greatest interest.

## 3. Materials and Methods

### 3.1. Site Description

**Figure 1:**
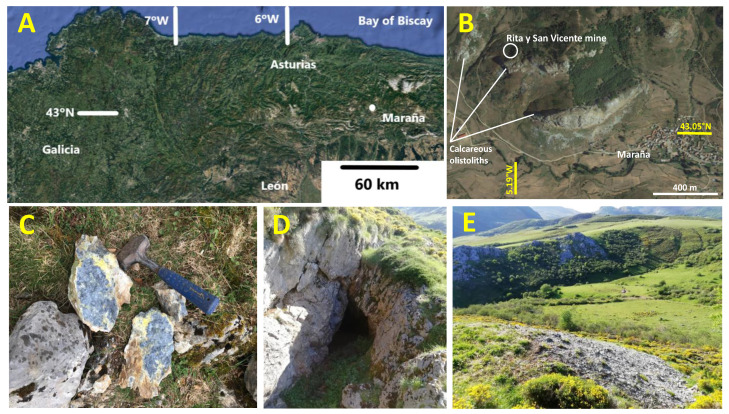
(**A**,**B**) Location of the Rita y San Vicente mine, NW of the locality of Maraña; (**C**) stibnite and Sb ochre mineralization; (**D**) upper mining works; (**E**) spoil heap.

The “Rita y San Vicente” mine is a small, abandoned Sb mine located in the NE of the province of León, 1.5 km NW of the village of Maraña (Figure 1; X_UTM_ = 322,415, Y_UTM_ = 4,768,883, UTM zone 30, European Terrestrial Reference System 1989).

The study area lies within the so-called “Pisuerga-Carrión unit” (Cantabrian Zone), just above a large fault that separates the Valdeón and Riaño domains. The Sb mineralization, which is considered to have been emplaced during the Permian distensional phase, shows typical features of hydrothermal origin deposits and is hosted in large calcareous olistoliths, which appear to be dispersed within a thick sequence of siltstones and shales locally known as the “Maraña Group” (Lower Stephanian).

A previous study [45] indicates an irregular morphology of the mineralization, combining both vein-type and stratiform Sb accumulations. There are several spoil heaps around the ancient mine site that contain mine wastes and low-grade ore and, which together constitute a volume of about 300 m^3^. There is no evidence of metallurgical activity in the vicinity of the former mine.

Despite being a small mine site, it was selected as a suitable case study based on (i) the carcinogenic nature of Sb, as well as the scarcity of studies on the environmental mobility of this metal; (ii) the simplicity of the mineralogy of the deposit, along with the absence of metallurgical works, eliminate interferences and allow for better interpretation of the results.

### 3.2. Mine Waste Sampling

The spoil heaps at the Maraña mine site are mainly composed of unmineralized limestone fragments (from workings carried out to access the ore body) and low-grade ore. Their study is critical for characterizing the mine wastes, which are the source of the soil pollutants. Both mineralized and unmineralized rock fragments were collected from the spoil heaps. These fragments are usually angular and decametric in size. The metallic mineralization is dominated by stibnite, usually dispersed within the hosted limestone in millimeter-sized crystals (see Figure 1C). After careful examination, representative samples were prepared for petrographic studies in the form of thin and polished sections. In particular, the geological features that influence the environmental dispersion of pollutants in a more decisive way are mineral textures (grain size, above all), ore geochemistry and host rock characteristics.

### 3.3. Soil Sampling

**Figure 2:**
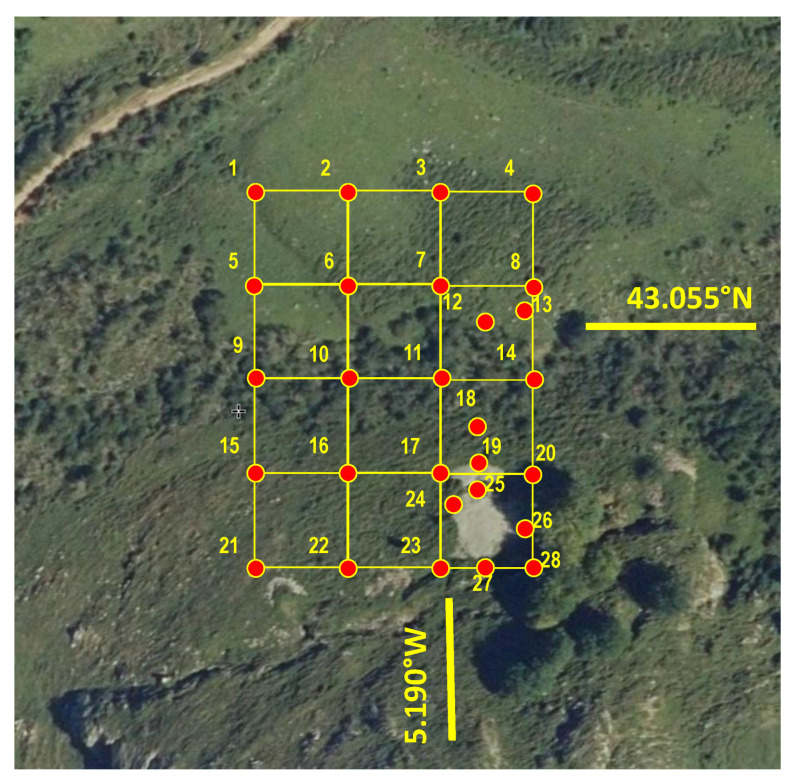
Soil sampling grid (scale: 25 m between samples in the regular grid).

A total of 28 soil samples were taken from the mine site -20 following a regular sampling grid (25 m between samples, see Figure 2; the maximum slope line is the NW-SE trend), and the remaining eight samples (nos. 12, 13, 18, 19, 24, 25, 26 and 27; Figure 2) were collected from selected points, at short distances downstream of the spoil heaps. All samples were taken using an Edelman auger (7 cm in diameter) at depths of up to 15 cm. The sampled surface is a steep area, with an average slope of 28%, and a combination of grassland and woodland with low scrub. The soil samples were air-dried (until constant weight) in the laboratory, disaggregated and manually ground in an agate mortar, and the fractions below 63 µm were retained for analysis.

### 3.4. Chemical Analyses (I): Sequential Extraction Procedure

As was previously stated, there is a great variety of sequential extraction procedures. One of the first and best known is that of Tessier et al. [46], which partitions elements into five operationally defined fractions: (i) exchangeable fraction (weakly sorbed species); (ii) fraction co-precipitated with carbonates (acid-soluble); (iii) fraction bound to Fe and Mn oxides (reducible); (iv) fraction incorporated into organic matter (oxidizable); (v) residual fraction (metals contained in the crystalline lattice of minerals).

Particular schemes have been created from the classical sequential extraction procedures, such as the BCR, designed to harmonize the diversity of SEPs [47]. The protocol proposed by Hall [48] was the one selected for this work. Mihaljevic et al. [49] pointed out that this method was the most suitable (among the four SEPs compared in their investigation) for As fractionation. The Hall [48] methodology obtains six fractions by the means of five leaching steps: (1) a demineralized water leach for extracting the water-soluble component, (2) 1 mol·dm^−3^ of ammonium acetate leach for exchangeable cations adsorbed by clay and elements co-precipitated with carbonates, (3) 0.1 mol·dm^−3^ of sodium pyrophosphate leach for elements adsorbed by organic matter, (4) 0.1 mol·dm^−3^ of hydroxylamine hydrochloride leach for elements adsorbed by amorphous Mn hydroxide, (5) 0.25 mol·dm^−3^ of hydroxylamine hydrochloride leach for elements adsorbed by amorphous Fe hydroxide and more crystalline Mn hydroxide. The remaining fraction is considered here as the residual one. Each fraction was analyzed by ICP-MS at Bureau Veritas Laboratories in Vancouver (Canada). The extracting agents and operating conditions used are those described by Hall [48].

Two groups of elements were considered within the context of this work. Firstly, As, Cu, Pb, Sb and Zn were selected as soil pollutants, their origin being related to migration from mine wastes. On the other hand, Fe-Mn, Ca-Sr and K were also considered to play an important role; Fe and Mn are mainly present in soils as oxides/hydroxides, constituting the basis of fractions 4 and 5. Ca is a relevant element in soil mineral fractions, as it is present in carbonate minerals and some clay species. Sr, in turn, is geochemically closely related to Ca. Finally, similar to Ca, K is also an essential element in the cationic group of clay minerals.

### 3.5. Chemical Analyses (II): Bulk Sample Analysis

Elemental analysis of the total contents was carried out by X-ray fluorescence combined with energy dispersive spectrometry (EDXRF) using a Niton Xl3t GOLDD+ instrument (50 kV, 20 µA, silicon drift detector, Thermo Scientific, Munich, Germany). The duration of each measurement, following the manufacturer’s recommendations, was 120 s.

### 3.6. Chemical Analyses (III): Energy Dispersive X-ray Spectrometry

Examination of the samples was performed by the scientific-technical services of the University of Oviedo using electron microscopy techniques with both scanning (20 kV) and transmission (200 kV) units -JEOL 6610LV and JEOL-JEM 2100F (JEOL, Tokyo, Japan), respectively. Both devices had an integrated energy-dispersive X-ray microanalysis module, with a resolution of 125 eV. The selected X-ray emission lines were K_α1_ for As, Cu and Zn, M_α1_ for Pb and L_α1_ for Sb.

The samples selected to be studied under electron microscopy were those with higher contents of Sb and other pollutants (those that lie outside of the regular grid in Figure 2, i.e., nos. 12, 13, 18, 19, 24, 25, 26 and 27).

## 4. Results and Discussion

### 4.1. Mine Wastes

**Figure 3:**
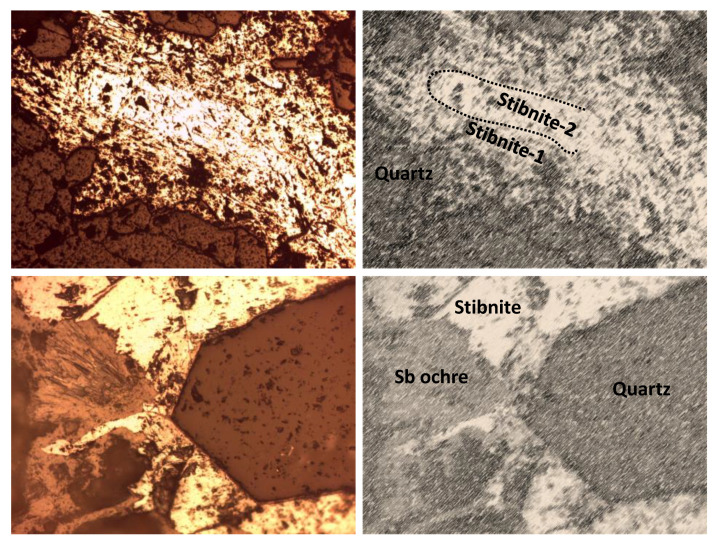
(**Above**): Typical texture of the primary mineralization—quartz and stibnite (two generations) filling open cavities within the host limestone (width of view: 2.6 mm). (**Below**): Detail of mineral replacement—supergene oxidation of stibnite causes Sb ochre growth (width of view: 650 µm).

The study of the mineralized samples leaves no doubt about the epigenetic character and hydrothermal origin of the ore. The primary mineralization was mainly composed of stibnite, which appeared in different open-space filling textures. Generally, the stibnite crystals were above 1 mm in diameter. Although mineral zoning is not an obvious feature, frequent stibnite overgrowth indicates the existence of superimposed hydrothermal events. The stibnite was accompanied by early and idiomorphic quartz crystals from hydrothermal origin, whose grain size did not usually exceed 0.5 mm. Concerning primary mineral species, only small (<100 µm) and scarce pyrite crystals were observed. Finally, it is noteworthy that most of the stibnite crystals showed moderate weathering, which is responsible for the neoformation of Sb oxides (Sb ochres) in thin rims, discontinuously following the outer outline of the stibnite crystals. All these features are shown in Figure 3.

On the other hand, the host rock was a medium-size (0.2 km^2^) limestone olistolith, surrounded by a thick layer of Stephanian siltstones and lutites. Thin-section examination of this rock highlighted a microcrystalline texture, where calcite is the main mineral species, appearing as equigranular microcrystals of 20 to 30 µm in size. Weak evidence of silicification and dolomitization can also be observed.

### 4.2. Soils (I): Whole Sample Chemical Analyses

**Figure 4:**
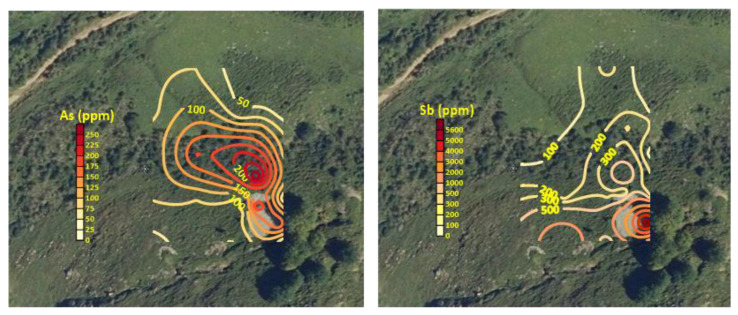
Geochemical maps showing the spatial distribution of the concentrations of As and Sb concentration (mg kg^−1^) in the sampled area (horizontal framing: 190 m).

The results of the analysis of the total content (average, minimum and maximum values) of some selected elements are shown in Table 1. It was assumed that Fe and Mn share a dual source, with both natural and anthropogenic origins. Sb, As, Cu, Pb and Zn can be considered elements whose presence in soil is related to mining activities. Among them, Sb and Pb show the highest concentrations, and As and Zn levels are also of concern. Most of the contents were above the European median concentrations for topsoil suggested in the Geochemical Atlas of Europe [50] (0.60 mg kg^−1^ Sb, 22.6 mg kg^−1^ Pb, 13 mg kg^−1^ Cu, 52 mg kg^−1^ Zn and 7.03 mg kg^−1^ As). The enrichment factors over the cited reference baseline value were 2281, 21, 3, 45 and 4 for Sb, As, Cu, Pb and Zn, respectively. The range of Sb content in European soils according to this source is 0.02–31.1 mg kg^−1^ Sb, whereas Kabata-Pendias [51] proposed the range of 0.25–1.04 mg kg^−1^ Sb for unpolluted soils. The local background value for Sb can be estimated at 3.3 mg kg^−1^, corresponding to a reference sample taken 400 m away from the mining site and free from its influence. The Spanish regulations on contaminated soil have established the generic reference levels of a contaminating substance in the soil as its concentration that does not entail a risk greater than the maximum level acceptable for human health or ecosystems. In the absence of generic reference levels defined for the province of León, where the mine is located, those defined for the neighboring province of Asturias are considered applicable for comparison, given their similar geological substrate. The soil sampled at the mine site had concentrations clearly above the reference levels in Sb (295 mg kg^−1^) and above those for As (200 mg kg^−1^) and Pb (800 mg kg^−1^) in some cases, if the scenario of industrial use is considered. However, it must be taken into account that there is livestock activity in the area and, although the residential scenario is not considered since the closest town (Maraña) is 1.5 km from the mine, there are nearby buildings, so there is some transit through the mining area. The elements related to the mineralization also show rather high relative dispersions, typical of an erratic spatial distribution. The spatial geochemical distribution shows, as expected, the highest values in the vicinity of the spoil heaps and a preferential migration downwards, roughly following the maximum slope line, as shown in Figure 4 (performed with QGIS) for As and Sb.

The average percentages from the total content of selected elements found at each step of the sequential extraction procedure are also presented in Table 1. As it was previously stated, the remaining percentage up to 100% (the majority) corresponds to the residual fraction. It can be easily deduced that, among the elements of anthropogenic origin, Sb is the least mobile, as it shows the highest percentage associated with the residual fraction (90.14%). On the contrary, Pb is the most mobile since its residual fraction represents 55.67% of the total. These values suggest that mechanical dispersion predominates over chemical dispersion. Regarding pollutants behavior in relation to the sequential extraction procedure, three different patterns can be distinguished:

(i) Cu and Sb were mainly extracted within the third leach (the fraction ideally bound to organic matter), being almost negligible in the rest of the fractions from the quantitative point of view.
(ii) As behaves in a different way: although Fraction 3 is also the predominant, the percentages of this metalloid leached in Steps 4 and 5 (the fractions bound to Mn and Fe oxides) are more significant than those obtained for Cu and Sb.
(iii) Finally, Pb and Zn are mainly leached-in similar quantities-in Steps 3, 4 and 5, providing Leach 2 (fraction bound to carbonates) not negligible percentages of the total concentration.

Assuming the proposal of Hall [48] in assigning each of the fractions to a certain mode of occurrence, the elements considered soil pollutants are predominantly linked to soil organic matter (Fraction 3) in all cases, except Zn, for which a slightly high percentage is associated with Fe-Mn oxides. This interpretation, a priori, could fit well with the widely described affinity of humic and fulvic acids of organic matter to retain pollutants in ionic form by adsorption, complexation and/or chelation. Fractions 1 and 2 would ideally represent the water-soluble components (Leach 1) and exchangeable cations adsorbed by clay and elements co-precipitated with carbonates (Leach 2). On the other hand, most of the Ca was mobilized within the ammonium acetate leach, indicating that this reagent is suitable for carbonate dissolution. However, it did not seem to have such an effect with clays, since K recovery in the leachate was very poor (0.92%). There are also some doubts about the effectiveness of 0.25 mol·dm^−3^ NH_2_OH·HCl to attack Fe oxides, with a modest 13.16% of the total Fe recovered in Leach 5. This reagent appeared to adequately solubilize Mn (38.34% in Leach 4 and 10.12% in Leach 5). A remarkable percentage of Mn was recovered in Leach 3. In this regard, Hall et al. [52] explain an analogous situation by suggesting a probable dispersion of Mn-containing colloidal particles (instead of Mn binding to organic matter).

### 4.3. Soils (II): Particle-Scale Micro-Analyses

**Figure 5:**
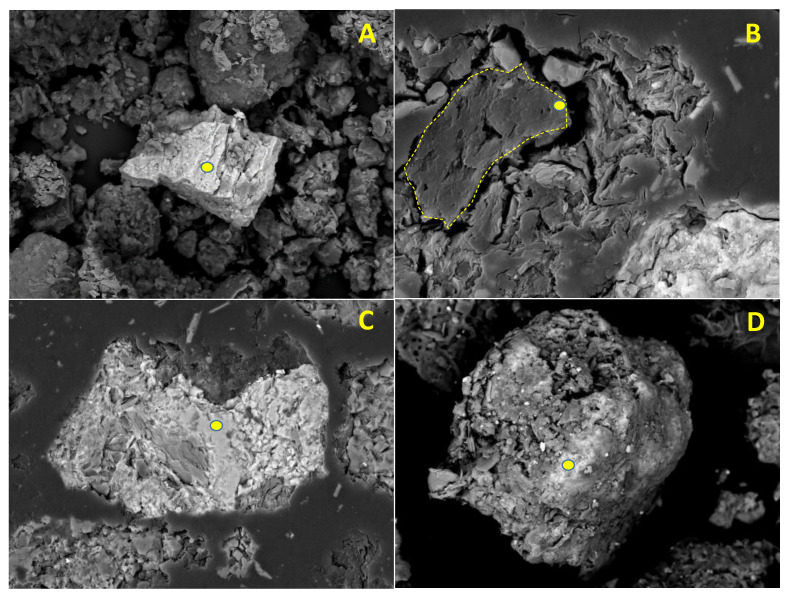
Different types of soil particles examined by means of backscattered electron (BSE-SEM) detection. Yellow points indicate areas of microanalysis. (**A**) Sb ochre (horizontal width: 100 μm, 64.65% Sb, 27.72% O, minor contents of Ca, Si and Al); (**B**) Organic matter (grain marked with yellow dashed line, horizontal width: 75 μm; 66.69% C, 20.18% O, 6.24% Si, 4.13% Al, 2.13% K, Mg, Ca and Fe below 1%); (**C**) Fe oxide (horizontal width: 150 μm, 54.49% Fe, 43.95% O, 1.34% Sb, 0.22% As); (**D**) Fe-Mn oxide (horizontal width: 110 μm, 40.70% O, 24.25% Fe, 12.06% Mn, 7.72% Si, 5.90% Al, 5.76%, Pb, 1.27% K, 1.00% Sb, Ca and As below 1%).

Instrumental methods that allow grain-scale elemental analysis are very useful tools. Their results can be contrasted with those obtained by SEPs. Thus, some representative samples (see the explanation in the last paragraph of Section 3) were mounted over a conductive carbon adhesive tape and studied by scanning electron microscopy-energy dispersive X-ray spectrometry (SEM-EDX) under the conditions detailed in Section 3. This instrumental method, employing backscattered electron imaging, enables the quick identification of organic matter (low molecular weight), Fe-Mn oxides/hydroxides and Sb-bearing phases. The morphological features of the soil particles and the results of the microanalysis carried out on their surfaces allowed for extracting a series of deductions that are discussed following the images shown in Figure 5.

Figure 5A shows the appearance of the Sb ochres, which, as expected, were the most abundant group among the Sb-containing particles. They usually had angular morphologies and diameters up to 75 µm (grain sizes were measured by means of Image-J software from original BSE-SEM images). In most cases, these particles usually have small sheets of lighter minerals, possibly undifferentiated phyllosilicates, bonded to their surfaces. The presence of oxyplumboromeite (Pb_2_Sb_2_O_6_O) was also observed. Organic matter particles (Figure 5B, central particle), are relatively abundant, usually found in rounded—sometimes highly complex—shapes and somewhat larger sizes when compared to Sb-ochres (several tens of microns). Numerous microanalyses were performed on the cleanest surfaces of these particles and the obtained results indicate no As-Cu-Pb-Sb-Zn presence, so it can be concluded that these elements are not really linked to the organic matter, at least above the theoretical resolution of the technique (0.1%). Figure 5C,D shows the typical aspect of Fe and Fe-Mn oxides, respectively. When microanalysis was performed on the surface of these soil components, variable amounts of Sb and Pb were always detected (up to 1.84 and 5.76%, respectively), as well as As in many cases (in contents close to the detection limit of the method).

It is therefore unquestionable that the amounts of Pb, Zn, As and Sb recovered in the third fraction are not really associated (at least, for the most part) with organic matter. They were leached by 0.1 mol·dm^−3^ sodium pyrophosphate (accepted as the most specific solvent for humic and fulvic substances), and a possible explanation for this apparent contradiction is reported by Hall et al. [52]. These authors point out that the dispersion of fine-grained inorganic colloidal material can contribute to metal liberation with a pyrophosphate attack. They also state that a small attack on sulfides or Fe oxyhydroxides could take place under certain conditions. On the contrary, and especially for Sb, it can be concluded that the amount of elements adsorbed on Fe-Mn oxides and hydroxides seems to be higher than the contents resulting from sequential extraction determinations (Fractions 4 and 5). When working with EDX, the proper selection of the areas of analysis is very critical. The surfaces must be free of interfering materials; this fact is clearly shown in Figure 5B,C, where very small bright particles, presumably from Sb-bearing minerals (ochre and antimonite, in order of importance), can be seen finely scattered over many areas. As for As, the stable form of Sb in aerobic systems is Sb (V), and its immobilization by Fe (hydr) oxides is widely described in the scientific literature (e.g., [53]).

**Figure 6:**
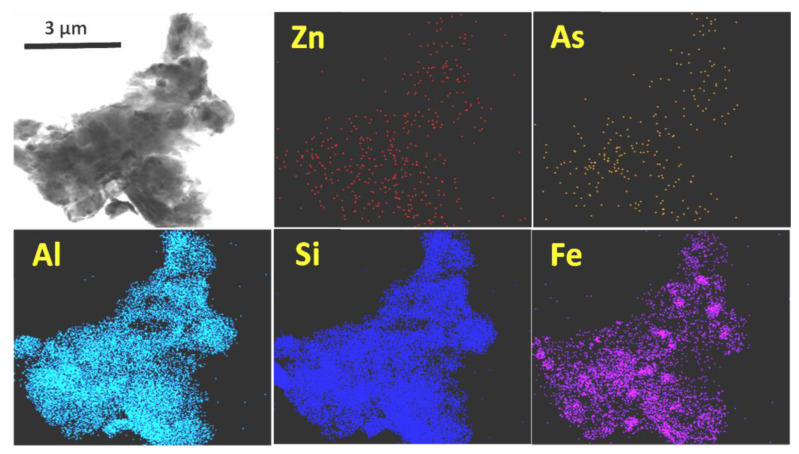
Zn, As, Al, Si and Fe distribution maps in a particle (first image) of a clay mineral.

Finally, by means of high-resolution transmission electron microscopy-energy dispersive X-ray spectrometry (HRTEM-EDX), multi-elemental geochemical maps were made of several clayey particles, such as those shown in Figure 6. On all clay surfaces, As was detected (0.08–0.47%) and in most of them, Pb was also found (up to 0.53%). Zn, despite not being numerically quantified in the EDX results, did appear as a faint stippling on the map. On the contrary, neither Sb nor Cu were detected on the surface of the clay particles. Although As and Zn were distributed throughout the entire clay surface, they tended to concentrate to a greater extent in the Fe-rich zones. Considering the relative abundance of clay minerals in the soil, and the distribution of As, Cu, Pb, Sb and Zn in the fractions detailed in Table 1, the following may be deduced:

(a) For Pb (usually present in very scarce amounts on clay surfaces), Zn (detectable, but not quantifiable) and Sb (undetectable), the cross-observations of sequential extraction and microanalysis fit acceptably well.
(b) Conversely, in relation to As, the results of both methods do not adequately match. As adsorption on the surface of clay particles should be, at least, quantitatively similar to that detected to be bound to Fe-Mn oxides and hydroxides, according to observations derived from SEM and TEM studies. The microanalysis results show that both, clays and Fe-Mn oxides contain similar amounts of bounded As, whereas the sequential extraction results suggest that the As associated with Fe + Mn oxides is an order of magnitude greater than the As contained in the clay fraction, despite the fact that the clay content in the soil far exceeds that of Fe + Mn oxides. The use of ammonium acetate seemed to be suitable for carbonate dissolution and for cation exchange capacity (CEC) determination in clays but did not seem to achieve complete solubilization of the As bound to clay surfaces. Some previous works have already indicated the underestimation of sequential extraction procedures in the role played by clays in As retention in soils [38].

## 5. Conclusions

After considering all of the above sections, the following general conclusions can be drawn:

- In the case study presented in this work, pollutants of concern (Sb, As, Cu, Pb and Zn) that occurred in the soil samples were mainly present in the residual phase (from 55.67% for Pb to 90.14% for Sb), that is, forming part of the mineral crystalline structures in their own primary or secondary minerals. Thus, they were not mobilized from an environmental-geochemical perspective. This is a frequent and common feature, especially in cases of extreme pollution. Environmental mobility decreases in the following order: Pb > Zn > Cu > As > Sb, assuming the mobility criterion is the percentage of each element that has been removed from the original source.
- Besides forming part of specific minerals, most of these elements were extracted with sodium pyrophosphate and, consequently, ideally bound to organic matter (from 6.71% for Zn to 18.90% for Cu). The latter consideration does not match well with the SEM-EDX examination of the samples and seems to indicate that the used reagent is capable of solubilizing metals and metalloids present in other forms, apart from binding to organic matter.
- Results of microanalyses carried out on particles of Fe and Fe-Mn (hydr)oxides match well for As, Pb and Zn with the percentages of these elements leached in steps 4 and 5 of the applied SEP. On the contrary, this solvent is not optimal to evaluate the role played by Fe-Mn (hydr)oxides in Sb fixation.
- Since As was detectable (0.1–0.5%) in most of the microanalyzed clay surfaces, ammonium acetate did not seem to be able to dissolve the amount of this metalloid associated with this type of minerals (only 0.56% of the total was recovered).
- Sequential extraction techniques are a useful tool in environmental studies and can contribute to a better understanding of the behavior of the chemical elements of greatest interest. However, the interpretation of the results of these tests in terms of the target phases or physical mechanisms of retention should be interpreted with caution.

## Figures and Tables

**Table 1:** Column 2: Average content of selected elements in the sampled soils. Columns 3–7: Average extraction percentages from total content obtained in each step of the SEP. Ranges of values are indicated in the brackets.

	Mean Content		Extraction Percentages from Total Content Obtained in Each Fraction (%)				
			Leach 1	Leach 2	Leach 3	Leach 4	Leach 5
As	145.3 (65.6–271.2)	mg kg^−1^	0.34 (0.18–0.70)	0.56 (0.12–1.85)	9.63 (6.70–17.11)	2.43 (1–5.71)	6.46 (1.85–13.93)
Cu	40.4 (25.2–112.8)	mg kg^−1^	0.55 (0.21–1.67)	0.75 (0.26–1.85)	18.90 (7.27–29.93)	1.45 (0.43–3.10)	0.57 (0.24–0.82)
Pb	1027.9 (13.5–7762.4)	mg kg^−1^	0.12 (0.03–0.29)	4.46 (1.69–9.07)	15.69 (7.05–32.01)	12.01 (4.95–38.56)	12.05 (4.63–29.40)
Sb	1368.4 (196.4–5775.9)	mg kg^−1^	0.36 (0.17–0.87)	0.21 (0.11–0.48)	8.54 (1.91–17.24)	0.22 (0.13–0.49)	0.53 (0.35–1.06)
Sr	80.1 (68.1–99.2)	mg kg^−1^	0.44 (0.13–1.61)	9.88 (5.72–15.92)	2.57 (1.34–5.25)	2.82 (1.14–4.24)	0.73 (0.29–1.04)
Zn	207.5 (93.9–637.7)	mg kg^−1^	0.22 (0.07–0.90)	3.20 (0.57–5.08)	6.71 (3.42–13.94)	6.61 (2.43–17.73)	7.43 (4.88–12.99)
Ca	0.97 (0.4–1.63)	%	2.18 (0.95–3.90)	51.23 (37.01–82.82)	12.16 (7.72–15.80)	4.87 (3.42–6.63)	1.44 (0.67–2.99)
Fe	2.62 (1.96–2.89)	%	0.08 (0.06–0.14)	0.03 (0–0.10)	7.41 (4.71–12.97)	3.95 (2.97–4.62)	13.16 (9.73–15.99)
K	1.32 (1.05–1.76)	%	0.41 (0.09–1.84)	0.92 (0.45–2.15)	0.87 (0.45–1.76)	0.21 (0.16–0.24)	0.27 (0.20–0.43)
Mn	1467.7 (853.4–1989.8)	mg kg^−1^	0.30 (0.07–1.44)	8.64 (3.86–23.52)	37.45 (17.69–60.81)	38.34 (13.08–67.10)	10.12 (5.03–15.39)
